# Spatial and age-related changes in the microstructure of dystrophic and healthy diaphragms

**DOI:** 10.1371/journal.pone.0183853

**Published:** 2017-09-06

**Authors:** Catherine C. Henry, Kyle S. Martin, Bridget B. Ward, Geoffrey G. Handsfield, Shayn M. Peirce, Silvia S. Blemker

**Affiliations:** 1 Department of Biomedical Engineering, University of Virginia, Charlottesville, Virginia, United States of America; 2 Auckland Bioengineering Institute, University of Auckland, Auckland, New Zealand; 3 Department of Ophthalmology, University of Virginia, Charlottesville, Virginia, United States of America; 4 Department of Mechanical and Aerospace Engineering, University of Virginia, Charlottesville, Virginia, United States of America; Universitat Wien, AUSTRIA

## Abstract

Duchenne muscular dystrophy (DMD) is a progressive degenerative disease that results in fibrosis and atrophy of muscles. The main cause of death associated with DMD is failure of the diaphragm. The diaphragm is a dome-shaped muscle with a fiber microstructure that differs across regions of the muscle. However, no studies to our knowledge have examined spatial variations of muscle fibers in dystrophic diaphragm or how aging affects those variations in DMD. In this study, diaphragms were obtained from *mdx* and healthy mice at ages three, seven, and ten months in the dorsal, midcostal, and ventral regions. Through immunostaining and confocal imaging, we quantified sarcomere length, interstitial space between fibers, fiber branching, fiber cross sectional area (CSA), and fiber regeneration measured by centrally located nuclei. Because DMD is associated with chronic inflammation, we also investigated the number of macrophages in diaphragm muscle cross-sections. We saw regional differences in the number of regenerating fibers and macrophages during the progression of DMD in the *mdx* diaphragm. Additionally, the number of regenerating fibers increased with age, while CSA and the number of branching fibers decreased. Dystrophic diaphragms had shorter sarcomere lengths than age-matched controls. Our results suggest that the dystrophic diaphragm in the *mdx* mouse is structurally heterogeneous and remodels non-uniformly over time. Understanding regional changes in dystrophic diaphragms over time will facilitate the development of targeted therapies to prevent or minimize respiratory failure in DMD patients.

## Introduction

Duchenne muscular dystrophy (DMD) affects 1 in 3,500 live male births and results from an x-linked gene mutation that causes a deficiency of the protein dystrophin [1–3]. Loss of dystrophin results in progressive damage of muscle, as evidenced by increased fibrosis, branched muscle fibers, chronic inflammation, muscle fiber atrophy, and fatty infiltration [4–8]. Ultimately, respiratory failure is the leading cause of death in DMD patients, which is largely a result of degeneration and associated weakening of the diaphragm muscle [3,9]. In order to understand the age-related progression of diaphragm degeneration, it is important to develop a greater understanding of diaphragm microstructure in DMD and how microstructure changes with age.

It is known that diaphragm microstructure changes as a result of the aging process in both healthy and dystrophic diaphragms. Healthy, aging diaphragms undergo muscle weakening and atrophy and exhibit decreased cross sectional area (CSA) of type IIx/IIb fibers [10]. In contrast to these healthy age-related changes, diaphragms of the *mdx* mouse, a widely used mouse model of DMD [8,11,12], display varied muscle fiber size, necrosis, and an increase in connective tissue after only 6 months [8]. Connective tissue continues to increase and fibrosis develops by 270 days (~9 months) [13]. By this time, the *mdx* diaphragm also displays significant fiber CSA decreases compared to controls. In *mdx* diaphragms, slow myosin is doubled relative to controls at 16 months, a phenomenon also documented in DMD [8,14]. By 16–22 months of age, the optimal fiber length in *mdx* diaphragms is shorter than age-matched controls and, at 24 months, *mdx* diaphragms have seven times the collagen density of controls [8]. Due to the dramatic microstructural changes in *mdx* diaphragms over time, age is an important factor to consider when studying DMD [8,13].

In addition to temporal changes, the microstructure of healthy diaphragms also varies spatially. Muscle fiber area, perimeter, major diameter, and shape are dependent on the location between the central tendon and chest wall where the measurement is obtained [15]. Regional differences in neuromuscular junction spacing, metabolism, diaphragm thickness, and muscle fiber length and type, have also been observed [15–20]. Although the structural properties of the diaphragm are variable both with age and location, no studies conducted to date have examined regional differences in dystrophic diaphragms or how the aging process affects regional differences in dystrophic muscle microstructure. In order to better understand the spatial variation in dystrophic diaphragms and the microstructural changes that give rise to disease progression, a thorough investigation of healthy and dystrophic diaphragm microstructure is needed.

The purpose of this study is to test the overarching hypothesis that there is spatial heterogeneity in the remodeling of dystrophic diaphragms during aging. To do this, we quantified sarcomere length, interstitial space between muscle fibers, muscle fiber branching, number of fibers with centrally located nuclei, and fiber CSA in three different regions of diaphragms obtained from healthy and *mdx* mice. Because DMD is also associated with chronic inflammation, we quantified the number of macrophages in histological muscle cross-sectional specimens from each of the three regions. These metrics were examined in *mdx* and healthy mice at three, seven, and ten months of age. The first signs of degeneration in the *mdx* diaphragm are seen around 30 days of age and the diaphragm progressively degenerates over time. We thus chose the three, seven, and ten month time points, because they represent the early, middle, and late stages of disease in the *mdx* mouse [8,21]. Healthy mice were used as a control to assess the effect of dystrophy on the diaphragm over time.

Our results support our hypothesis and suggest that there is non-uniform remodeling of the dystrophic diaphragm. Additionally, the unique and extensive data set that we present for healthy and dystrophic murine diaphragms describes numerous microstructural characteristics of this muscle throughout the aging and disease time course. As a result, these data can serve at least two important purposes: 1) correlating different metrics across time points sheds light on potential relationships between remodeling mechanisms (e.g. inflammation and fibrosis), and 2) parameterizing and/or validating mechanistic computational models of disease progression using these data can identify causes of disease and predict the effectiveness of new drugs for treating DMD [22–24].

## Materials and methods

### Mice and study overview

All animal use protocols related to the research were approved by the University of Virginia’s Institutional Animal Care and Use Committee (PHS Assurance number #A3245-01; USDA registration #: 52-R-0011). Thirty-eight total male C57BL/10ScSn-Dmd^*mdx*^/J (*mdx*) and C57BL/10ScSn (control) mice at three (n = 13 of each group), seven (n = 3 of each group), and ten (n = 3 of each group) months of age were purchased from Jackson Laboratory (Bar Harbor, Maine, USA).

### Diaphragm harvest and immunostaining

Mice were euthanized via carbon dioxide inhalation. The abdominal cavities of the mice were opened to equalize pressures between the abdominal and thoracic cavities. We made the assumption that the equalization of pressure would result in the diaphragm sarcomere lengths returning to optimal length or near optimal length as a result of balance of external and internal forces. The diaphragms were fixed *in situ* with 4% paraformaldehyde (PFA) in phosphate buffered saline (PBS) for five minutes. Afterwards, the diaphragms were surgically excised, stripped of fascia, and cut in half along the mid-sagittal plane. Based on previous findings, we regarded the two halves of the diaphragm as symmetrical in microstructure [15]. We randomly divided the halves into one of two groups for whole mounting or sectioning.

One half of the diaphragm was whole mounted, fixed for an additional five minutes with 4% PFA, and immunostained, as described below. The muscles were permeabilized in 0.2% saponin/PBS solution overnight. Diaphragms were incubated for one hour at room temperature in blocking solution (5% control goat serum (G9023 [Sigma-Aldrich, St. Louis, MO, USA]) in 0.2% saponin/PBS). The samples were stained overnight with monoclonal anti-α-actinin (A7811 [Sigma-Aldrich, St. Louis, MO, USA], 1:300) and anti-mouse collagen type I (AB765P [Millipore, Billerica, MA, USA], 1:200) diluted in blocking solution. The following day, the diaphragms were washed three times for ten minutes with 0.2% saponin/PBS. After washing, the samples were stained for two hours at room temperature with Alexa Fluor 546 and 647 (A11018 and A21246 [Invitrogen, Carlsbad, CA, USA], 1:400) diluted in blocking solution. The diaphragms were washed three times for ten minutes with 0.2% saponin/PBS solution.

**Fig 1:**
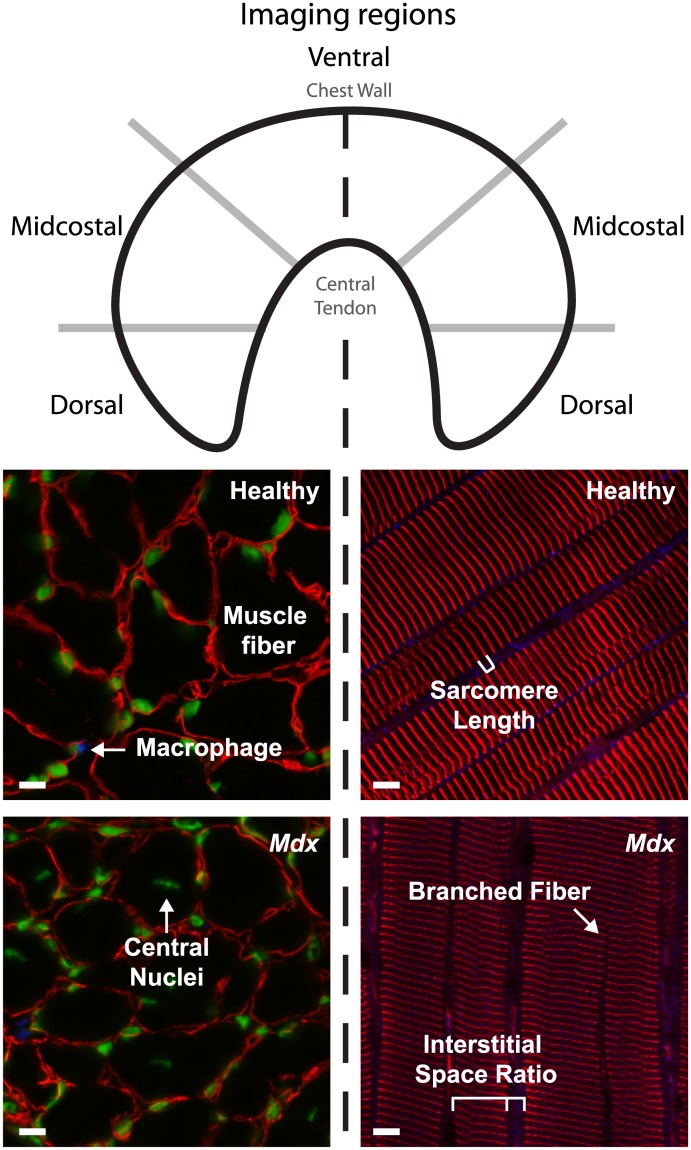
Experimental partitioning of the diaphragm to quantify regional variability in muscle microstructure. Top: Schematic representing the defined regions of the diaphragm. The diaphragm was split into two halves, bisecting the ventral region. Left: Histological cross-sections of the diaphragm were used to quantify CSA, regenerating fibers, and macrophages. Right: Whole mounted diaphragm was used to quantify sarcomere length, interstitial space, and branched fibers. Representative confocal images of the left (red = laminin, blue = CD68+ macrophages, green = nuclei) and right (red = alpha-actinin, blue = collagen I) halves of the immunostained diaphragms obtained from healthy and *mdx* mice (60x objective). Bar = 10 μm.

The other half of the diaphragm was divided into three regions: dorsal, midcostal, and ventral (Fig 1). The muscle regions were placed in optimum cutting temperature solution (23730571 [Fischer HealthCare, Houston, TX, USA) and rapidly frozen in liquid nitrogen cooled isopentane (M32631 [Sigma Aldrich, St. Louis, MO, USA]). The frozen samples were cut into ten-micrometer cross-sections with a cryostat (CM1950UV [Leica, Buffalo Grove, IL, USA]). The cross-sections were mounted on microscope slides and fixed for ten minutes with acetone and then washed twice for five minutes with PBS. The cross-sections were permeabilized for one hour in blocking solution (5% control donkey serum (D9663 [Sigma-Aldrich, St. Louis, MO, USA]) in 0.2% saponin/PBS) and then stained overnight with anti-laminin (L9393 [Sigma-Aldrich, St. Louis, MO, USA], 3:1000) diluted in blocking solution. The next day, the diaphragms were washed three times for ten minutes with 0.2% saponin/PBS. The muscle cross-sections were stained with conjugated rat anti-mouse CD68 (MCA1957A647T [Bio-Rad, Hercules, CA, USA], 3:1000), SYTOX green nucleic acid stain (S7020 [Life Technologies, Carlsbad, CA, USA], 1:50,000), and Alexa Fluor 546 (A10040 [Life Technologies, Carlsbad, CA, USA], 3:1000]. The diaphragm muscle cross-sections were again washed three times for ten minutes with 0.2% saponin/PBS solution.

### Imaging and image analysis

Digital images of immunolabeled diaphragms were acquired using confocal microscopy (Nikon, Melville, NY, USA; Model TE200-E2; 4x, 10x, 20x, and 60x objectives). Whenever possible, six or more 60x confocal images were taken in each of the three regions. Confocal images were taken sequentially along the chest wall and central tendon in each region. For each image, the XY position was adjusted along the central tendon/chest wall before focusing to a random Z position. These confocal images were used to quantify the following structural and cellular characteristics of the diaphragms: 1) sarcomere length, 2) interstitial space between muscle fibers, 3) branched muscle fibers, 4) muscle fiber CSA, 5) fibers with centrally located nuclei, and 6) macrophages. All data was analyzed using a blinded, trained observer, with the exception of the fibers with centrally located nuclei, which was analyzed by two blinded observers.

Sarcomere length: A script was written in MATLAB (R2013a, The Mathworks, Inc., Natick, MA) to determine average sarcomere length in each 60x image as follows. First, gray-scaled images were thresholded to create black and white images; next, a 2-dimensional fast Fourier transform was applied to the images. We band pass filtered the frequency image to remove frequencies corresponding to unphysiological sarcomere lengths (below 1.5 μm and above 4.5 μm). Modal sarcomere length was calculated from the maximum frequency in the first harmonic. The MATLAB script was validated using computer-generated idealized images with equally spaced sarcomere lengths and using muscle images from control and *mdx* mice, whose sarcomere lengths were measured by hand.

Interstitial space between muscle fibers: Analysis of the interstitial space between muscle fibers was accomplished using ImageJ (NIH, Bethesda, MD, USA) imaging software to measure the relative widths of the spaces between each muscle fiber in 60x confocal images (Fig 1). A reference line was drawn orthogonal to the muscle fibers along which fiber widths and interstitial widths were measured. The ratio of the width of the interstitial space to the width of the muscle fiber was then calculated.

Branched muscle fibers: Branched fibers were identified by visually tracking each muscle fiber in the image from one edge of the image to the opposite edge. Branching was evidenced by the presence of a “Y” in the fiber structure, or the apparent splitting of a fiber into two narrower fibers (“branches”) with sarcomeres that were parallel with the un-branched portion of the fiber (i.e. the “trunk”). The percentage of branched fibers per image was calculated by dividing the number of branched fibers by the total number of fibers in each image and averaging the percentage of branched fibers in the images of each region (Fig 1). At least 28 images were taken of each region for each age group.

Muscle fiber cross-sectional area: CSA was measured using ImageJ by outlining the muscle fibers, as indicated by the immunostain for laminin, in the 60x confocal images with the freehand selection tool. The minimum number of fibers analyzed in a region for a mouse type in an age group was 514 fibers.

Regenerating muscle fibers: Regenerating fibers were identified as muscle fibers with centrally located nuclei [25]. The percentage of regenerating fibers per field of view was calculated by dividing the number of regenerating fibers by the total number of fibers in the field of view.

Macrophages: Macrophages were identified by the presence of overlapping nuclei and macrophage (CD68) stains. The number of macrophages identified was divided by the total area of the field of view.

### Statistical analysis

All data were analyzed with Holm-Sidak comparison tests, which include family-wise error rate correction for multiple comparisons [26]. Within every measurement (e.g. sarcomere length), we analyzed every permutation of mouse type, age, and region. Although interdependencies between the metrics may exist, we were not able to make statistical comparisons between metrics due to lack of normality and unequal variances of variables, as well as the large number of variables within each metric. All statistical analyses were performed with SigmaStat (Systat Software Inc, Version 3.5, Germany). Statistical significance was asserted at P<0.05.

## Results

### *Mdx* diaphragms display maladaptations associated with dystrophy

**Fig 2:**
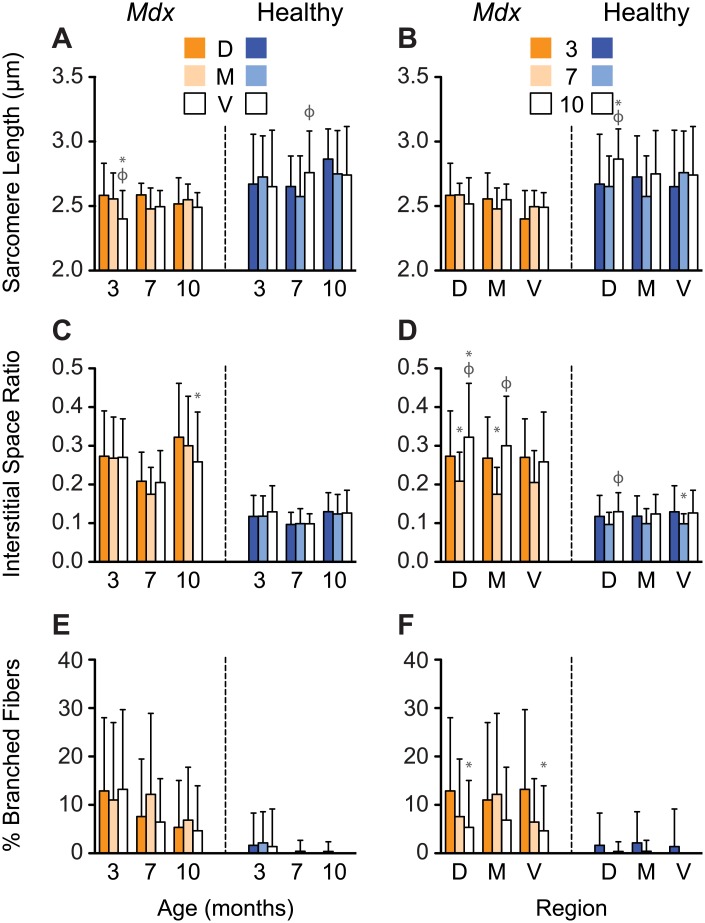
*Mdx* diaphragms exhibit a clear progression of muscular dystrophy as mice age. (A, C, E): Sarcomere length, the interstitial space ratio, and the percent of branched fibers are spatially heterogeneous in both the healthy and dystrophic diaphragm. Dystrophic diaphragms have shorter sarcomeres and more interstitial space and branched fibers than control mice. * = P<0.05 compared to dorsal, φ = P<0.05 compared to midcostal. (B, D, F): Regional differences in diaphragm microstructure are altered with age. Interstitial space varies with age, whereas the percent of branched fibers decreases with age in healthy and dystrophic diaphragms. * = P<0.05 compared to three months, φ = P<0.05 compared to seven months. D, M, V: dorsal, midcostal, and ventral regions. Values are means ± SD.

**Fig 3:**
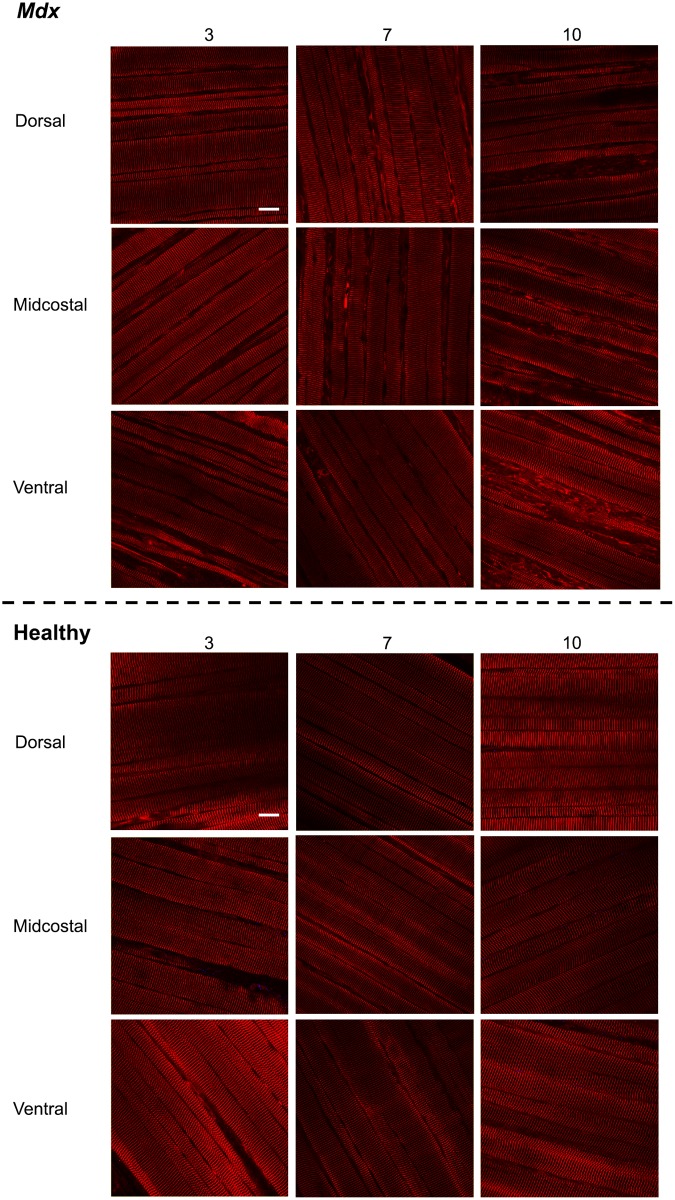
*Mdx* diaphragms have greater interstitial space and more branched fibers than healthy diaphragms. Representative confocal images (60x objective) of sarcomere length, interstitial space, and branched fibers in diaphragms from 3, 7, and 10 month old *mdx* and healthy mice in the dorsal, midcostal, and ventral regions (red = alpha-actinin). Bar = 25 μm.

**Fig 4:**
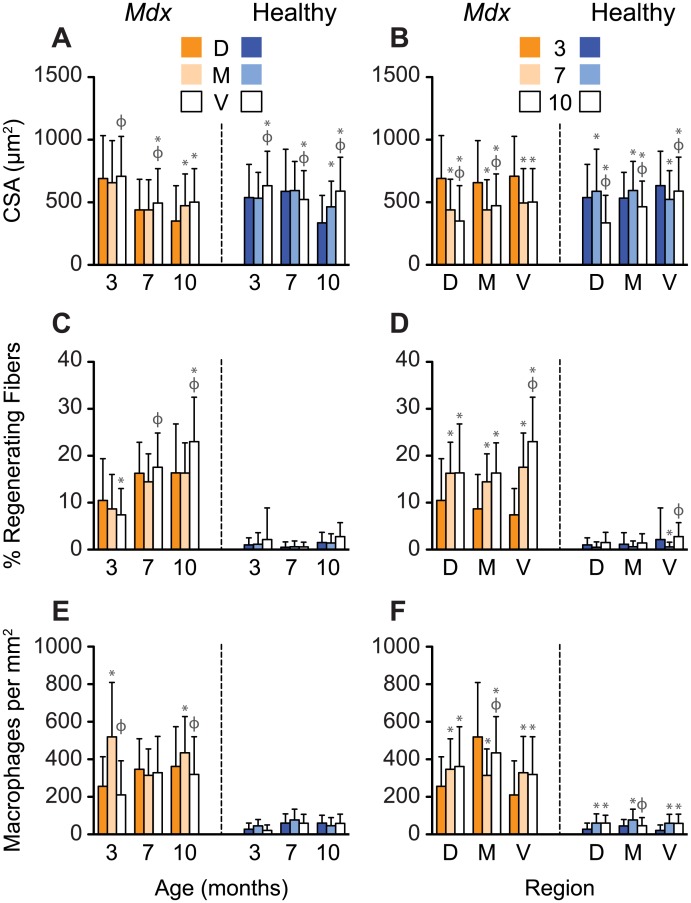
Healthy and dystrophic diaphragms are regionally diverse. (A, C, E): CSA, the percentage of regenerating fibers and macrophages exhibit spatial variations. Dystrophic diaphragms have a greater percentage of regenerating fibers and macrophages and smaller CSAs than controls. * = P<0.05 compared to dorsal, φ = P<0.05 compared to midcostal. (B, D, F): Regional differences in microstructure are altered with age. In three and ten month old *mdx* mice, the number of macrophages is increased in the midcostal region compared with the ventral and dorsal region. The number of regenerating fibers increases with age in the ventral region in *mdx* diaphragms. * = P<0.05 compared to three months, φ = P<0.05 compared to seven months. D, M, V: dorsal, midcostal, and ventral regions. Values are means ± SD.

**Fig 5:**
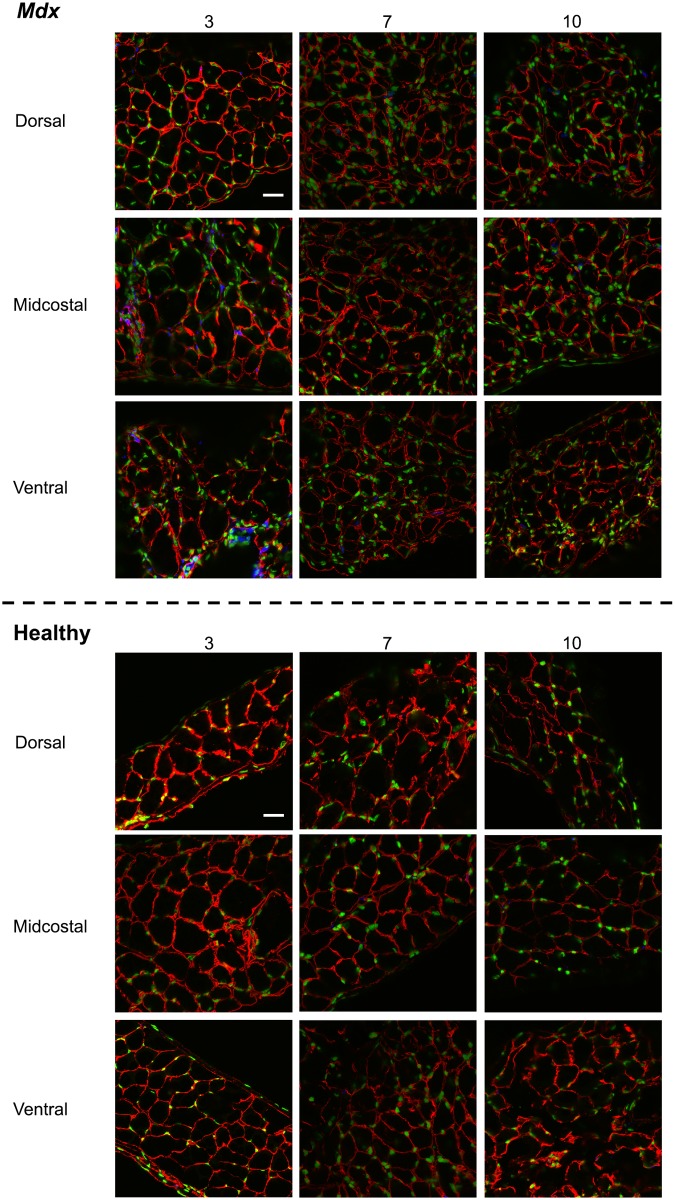
*Mdx* diaphragms have more regenerating fibers and macrophages than healthy diaphragms. Representative confocal images (60x objective) of CSA, regenerating fibers, and macrophages in diaphragms from 3, 7, and 10 month old *mdx* and healthy mice in the dorsal, midcostal, and ventral regions (red = laminin, blue = CD68+ macrophages, green = nuclei). Bar = 25 μm.

**Fig 6:**
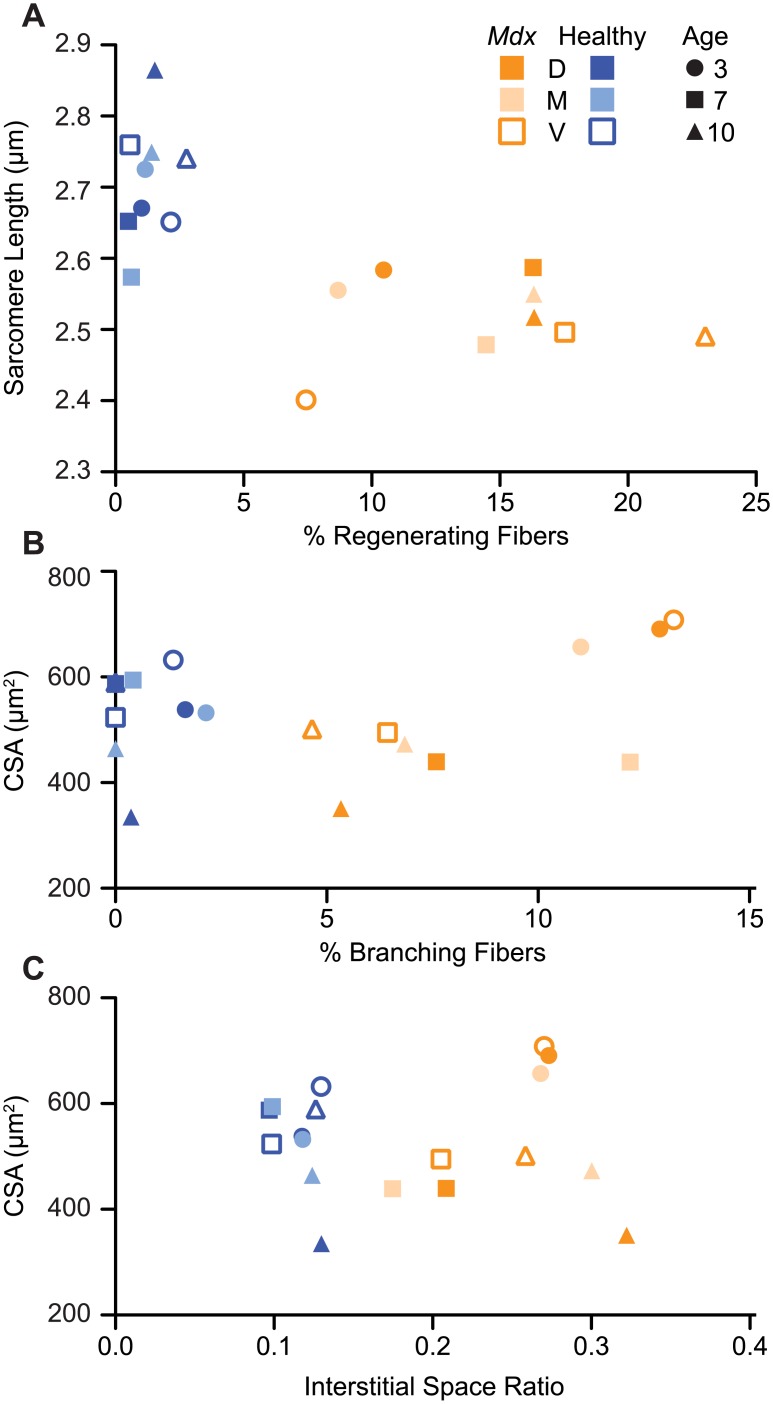
The microstructure of dystrophic diaphragms is dependent on region and age. (A): As the percentage of regenerating fibers increases, sarcomere length decreases. (B): In *mdx* diaphragms, the CSA and the percentage of branching fibers decreased with age. (C): When CSA is compared to interstitial space ratio, the age groups cluster together. D, M, V: dorsal, midcostal, and ventral regions. For each age group of healthy and *mdx* mice, the mean of each region is plotted for two metrics.

Dystrophic and healthy diaphragms had significantly different interstitial space ratios (0.26±0.12 vs 0.12±0.52; Figs 2C and 3) and percentages of fibers with centrally located nuclei (14.83±8.97 vs 1.29±2.83 percent; Figs 4C and 5). These differences were present at every observed age (P<0.001). Additionally, we observed statistically significant differences in sarcomere length (2.70±0.35 vs 2.53±0.19 μm), the percentage of branching fibers (1.03±5.11 vs 9.48±13.91 percent), macrophage counts (51.97±46.82 vs 345.15±213.63 per mm^2^), and CSA (530.07±260.45 vs 502.37±296.32 μm^2^) between healthy and *mdx* diaphragms (P<0.001, Figs 2–5). With increases in the percentage of fibers with centrally located nuclei, we observed decreases in sarcomere length, a trend that was independent of both age and region (Fig 6A).

### Muscle microstructures of the diaphragm adapt with age

*Mdx* diaphragms exhibited increases in the percentage of regenerating fibers and there were age-related decreases in CSA and fiber branching. The percentage of regenerating fibers in the *mdx* diaphragm increased by 85.1% from three to seven months (P<0.001) and 14.3% from seven to ten months (P = 0.002) (Fig 4C, S1 Table). We found decreases with age for muscle fiber CSA (three, seven, and ten months: 683.98±334.53, 457.37±254.91, and 442.33±276.82 μm^2^) and for the percentage of branching fibers (three, seven, and ten months: 12.13±15.71, 8.79±13.10, and 5.70±10.02 percent) in *mdx* diaphragms (Fig 6B). The amount of branching decreased with age in control mice (three, seven, and ten months: 1.79±6.78, 0.14±1.32, and 0.12±1.16 percent; Fig 2E). The diaphragms in control mice at ten months of age had significantly longer sarcomere lengths (three vs ten months P = 0.011, seven vs ten months P = 0.009) and smaller CSAs (three vs ten and seven vs ten months P<0.001) than those in the other age groups. The sarcomere lengths and CSA for control mice were 2.69±0.37 μm and 562.10±250.51 μm^2^ at three months, 2.66±0.30 μm and 566.05±264.32 μm^2^ at seven months, and 2.79±0.32 μm and 484.79±257.43 μm^2^ at ten months, respectively. In both *mdx* and control animals, there was a significant decrease in the interstitial space ratio at seven months (0.20±0.08 and 0.10±0.03) compared to both three (0.27±0.11 and 0.12±0.06; P<0.001) and ten (0.29±0.13 and 0.13±.05; P<0.001) months, respectively (Fig 2C). Comparing interstitial space to CSA resulted in a clustering of the diaphragm regions according to age in the *mdx* mice (Fig 6C).

### Diaphragm microstructure is spatially heterogeneous

We observed differences in muscle microstructures between the three regions of the diaphragm (ventral, midcostal, and dorsal). At ten months of age, the CSA for each region was significantly increased from dorsal to midcostal to ventral, except for the midcostal to ventral region in the *mdx* diaphragm. This increasing CSA was seen in both *mdx* (dorsal, midcostal, and ventral: 351.01±282.23, 488.34±243.74, and 509.59±271.94 μm^2^; P≤0.001 for all but the midcostal to ventral region) and control (dorsal, midcostal, and ventral: 335.25±220.58, 464.25±205.73, and 589.47±271.69 μm^2^; P<0.001) animals (Fig 4A). There was also a significant increase in the percentage of regenerating fibers with age in the ventral region of *mdx* mice, with a 136 percent increase from three to seven months (P<0.001) and a 31 percent increase from seven to ten months (P<0.001, Fig 4D). This trend was less pronounced in the other regions of the diaphragm (S1 Table). As with age, we saw non-linear region-specific differences in muscle microstructures. Macrophages in the midcostal region of *mdx* diaphragms were elevated at three (519.81±289.55 per mm^2^) and ten months (434.41±193.70 per mm^2^) of age, and were significantly elevated compared to the dorsal (three and ten months: 256.08±158.07 and 361.93±212.19 per mm^2^; P≤0.013) and ventral region (three and ten months: 210.17±182.32 and 319.24±201.02 per mm^2^; P<0.001) at these time points. However, all of the regions were similar at seven months of age (dorsal, midcostal, and ventral: 347.23±162.20, 314.24±141.28, 328.74±193.26 per mm^2^; Fig 4E).

## Discussion

Although much is known about the mechanism causing DMD, it is unclear how the disease creates the array of maladaptations observed in patients. The diaphragm is of extreme interest in the study of DMD, because respiratory failure is the most common cause of death [3,9]. Previous studies have shown that healthy animal and human diaphragms have regional diversity in both structure and function [15–17,27–31]. This regional variation changes with age [32–34]. Additionally, there is evidence that suggests that some regions of the diaphragm are more susceptible to disease and failure than others [29]. Motivated by these findings, we set out to examine whether regional differences exist in the microstructure of the dystrophic diaphragm and if those differences vary with age.

Our results show that there are spatial variations in the *mdx* mouse dystrophic diaphragm that change as a result of the aging process. We found significantly elevated numbers of macrophages in the midcostal region of three and ten month old *mdx* mice compared to the dorsal and ventral regions, which may indicate that the midcostal region has greater inflammation at these time points. In ten month old healthy and dystrophic mice, the CSA increased from the dorsal to midcostal and from midcostal to ventral regions. These increases in CSA were all statistically significant except for the increase from the midcostal to ventral region in the *mdx* diaphragm. In addition, our studies showed that the percentage of regenerating fibers in the ventral region of *mdx* mice increased significantly with age (Fig 4D). These findings suggest that muscle microstructure differs across diaphragm regions, with the ventral region retaining the greatest CSA.

Muscle fibrosis, atrophy, and inflammation are hallmarks of DMD that become more pronounced with age [4,5,13,35–38]. Although evidence shows that fibrosis increases in *mdx* and healthy diaphragms with age [39,40], we found that the interstitial space (the metric in our study that is most affected by fibrosis) was more transient: interstitial space decreased from three to seven months, followed by an increase at ten months. Similar to previous studies, we found that the CSAs of healthy diaphragm fibers decreased in old age [10]. This decrease in fiber CSA was consistent with the increase in interstitial space at ten months. Muscle atrophy, characterized by decreased CSA and increased fibrosis, is associated with inflammation in DMD [5,38,41,42]. As in previous work, our results showed that macrophages, which are indicative of inflammation, increased in the diaphragms of *mdx* mice in the late stages of the disease [37].

Branched fibers are thought to be mechanically weak regenerating fibers, which are prevalent in DMD patients [7,43–46]. In contrast to studies that found increased branching in the limb muscles of *mdx* mice with age [7,45,47], our studies showed that branching decreased in dystrophic diaphragms with age. This could be due to the unique, domed structure of the diaphragm. It is also possible that fibers with large CSAs branch more frequently, and as the fibers decrease in CSA, they become less susceptible to branching. Although branched fibers are thought to be in a regenerative state [43–45], we found that as *mdx* mice aged, the number of branched fibers decreased while the number of regenerating fibers increased. The increased number of regenerating fibers is consistent with the progression of muscle damage in *mdx* mice [48–50]. While we identified regenerating fibers by the presence of centrally located nuclei, it is important to note that there are alternative interpretations of the physiological meaning of centrally located nuclei [25,51], and our results should be considered in this context. Regarding branched fibers and macrophages, it is possible that sampling error could have been introduced when obtaining images of these features by imaging the same fiber in different Z planes. We took pains to limit this possibility by quantifying branched fibers in a single image and changing the XY position along the central tendon/chest wall before obtaining new images.

Our results showed that the *mdx* diaphragms had greater atrophy and inflammation than healthy diaphragms. Consistent with previous literature, there was an increase in interstitial space [8,39,52], branching [7,45], regenerating fibers [52–54], and macrophages [37,38] in dystrophic diaphragms compared to controls. Previous evidence has also showed that the *mdx* diaphragm has a smaller CSA than healthy diaphragm around seven months of age [13].

In addition to differences in composition, *mdx* diaphragm sarcomeres were shorter (2.53±0.19 μm) than those in healthy diaphragms (2.70±0.35 μm). This result suggests that the *mdx* diaphragm operates at lower lengths on the force-length curve compared to healthy diaphragms. In rat muscle, optimal fiber length has been measured as 2.5 μm [55]. Using this value as an estimate for mouse optimal fiber length, this would suggest that the *mdx* diaphragm operates on the ascending limb while the healthy diaphragm operates on the plateau/descending limb of the force-length curve [56]. It should be noted that our fixation preparation equalized the forces between abdominal and thoracic cavities, allowing the muscle to shorten to a length where passive muscle forces were zero. It is possible, then, that a passive force of zero occurs at a shorter sarcomere length in *mdx* compared to healthy mice. Nevertheless, these differences in operating range and fiber CSA suggest functional impairments in the *mdx* diaphragm consistent with observations of alterations in breathing tidal volume in *mdx* as compared to healthy mice [57]. Interestingly, Ashmore et al. [58] found that dystrophic chick wing muscles were longer than those of healthy chick wings, which suggest that adaptations in sarcomere length is likely species and/or muscle specific.

Indeed, our study can help provide context for prior studies where regional differences in microstructure were not considered. For example, Louboutin et al. reported that the CSA in the *mdx* diaphragm at 270 days was smaller than that of age-matched healthy diaphragms; however, the location(s) where the measurements were taken were not provided [13]. Our study suggests that those measurements were not obtained from the dorsal region of the diaphragm. Stedman et al. examined strips of 16–22 month old *mdx* and healthy diaphragms and reported that they had approximately the same sarcomere length [8]. In our study, sarcomere lengths were similar when measured in the dorsal regions of age-matched three and seven month old healthy and dystrophic mice, but sarcomere lengths were significantly different in the ventral region. Further, we found inflammation to be one of the most variable metrics across the diaphragm regions. When macrophages were assessed in three month old *mdx* mice, there was up to a 147 percent difference in the number of macrophages, depending on the region that was evaluated (Fig 4E). Therefore, it is critical that future assessments of *mdx* and control diaphragms are done within well-defined regions that are clearly reported.

Although the *mdx* mouse is the most commonly used animal model for studying DMD, there are numerous limitations inherent in studying the *mdx* diaphragms. Due to the small size of the *mdx* diaphragm, it is more difficult to see regional differences than in a dog or human diaphragm. To sidestep this limitation, we chose a regional, rather than a radial, investigation (i.e. chest wall vs. central tendon). While DMD disease progression is better demonstrated by the *mdx* diaphragm than by other skeletal muscles in the *mdx* mouse [8,13,59], it is difficult to know how the time scale of diaphragm degeneration in the *mdx* mouse maps to that of a human and discussion is warranted on how the field may overcome this difficulty. Additionally, aside from performing cadaveric analyses, studying human diaphragms with the extensive microstructural characterization that was performed here in *mdx* mice would require image-guided, invasive biopsies that are not feasible in practice. Therefore, despite its limitations, the *mdx* mouse model of DMD is still extremely useful in allowing thorough investigations that provide insights into the disease that could not be gleaned from less thorough human studies.

Another potential limitation of our study was the procedure for diaphragm fixation. It has been shown that the diaphragm exhibits regionally dependent differences in fiber lengths measured *in situ* at end tidal lung volume versus those measured *in vitro*. Thus, as with *in situ* studies of the diaphragm in general, our measurements of fiber length, CSA, and interstitial space may not be representative of *in vivo* values [60]. Although it is uncertain whether small regional changes were introduced into our samples by our fixation method, our process was consistent across all animals and, thus, we expect that all diaphragms would have been affected similarly. We, therefore, believe that the relative differences observed between healthy and dystrophic diaphragms in our study are representative of differences that exist *in vivo*. Further studies may illuminate whether more subtle effects were not captured here by our experimental protocol.

Our results suggest that *mdx* and healthy diaphragms undergo non-uniform remodeling. In healthy diaphragms, this could be due to regional differences in mechanics, blood flow and nerve innervation patterns. Studies have found that the dorsal and midcostal regions of rat diaphragms have greater blood flow than the ventral region during rest and exercise [30]. It has also been found that the dorsal and ventral regions of the diaphragm are innervated by the C6 and C5 roots of the phrenic nerve, respectively [61,62]. Differences in blood flow and innervation could indicate that greater work is being done by certain regions of the diaphragm and could contribute to non-uniform degeneration. Like Poole et al., our findings suggest that different regions of the diaphragm undergo different degrees of degeneration during disease, and this spatial heterogeneity should be further studied to determine if and how it contributes to disease progression [29]. Specifically, future studies should examine spatial differences in the microanatomy of the *mdx* diaphragm at time points beyond ten months of age [37]. The regional distributions of fiber type should also be studied in dystrophic mice, because it has been shown that fiber composition is affected by both physiologic and disease conditions [10,63–66].

While the data set presented in this study provides a spatially detailed, time-course description of the microstructure of the healthy and diseased murine diaphragm, it can also be leveraged by computational modeling to produce novel mechanistic insights into muscle regeneration and degeneration. Indeed, computational models rely heavily on quantitative experimental data, and models of tissue adaptation (in health and disease) are particularly reliant on time-course data. In this regard, the data presented herein is ideal for parameterizing and/or validating computational models of muscle adaptation [23]. For example, Virgilio et al. created a computational model to study how structural alterations in muscle fibers of DMD patients change their mechanical properties [22]. The CSA and interstitial space ratio found in our study could be used to inform the input parameters in the Virgilio et al. model and, therefore, provide more accurate predictions of mechanical alterations in the muscle over time.

Our findings are useful for future studies aiming to examine how microstructure affects the function of *mdx* diaphragms. The results of our study suggest that future experiments should compare identical regions when testing the effects of treatments or other interventions on disease progression. Additionally, the formalization of a standardized reporting scheme for different diaphragm regions would aid in the comparison of results from different studies. Our observation that diaphragms experience region-specific structural adaptations with aging motivates future studies aimed at determining whether and to what extent the functionality of the diaphragm changes during the lifespan of an animal, both in disease and healthy conditions. Further explorations into diaphragm structural and functional heterogeneities could help identify regions that are more susceptible to disease and pinpoint an optimal timing schedule for treatments. Ultimately, having a better understanding of the microstructural changes in the diaphragm during disease progression is likely to benefit the development of treatments to extend the lifespan and improve the quality of life of DMD patients.

## Supporting information

S1 Table Means and standard deviations for each metric. (XLSX)
